# Investigation of hydraulic fracturing-induced seismicity in the Haynesville Shale

**DOI:** 10.1007/s10950-025-10296-x

**Published:** 2025-05-19

**Authors:** James P. Verdon, Alexander D. G. Harris

**Affiliations:** School of Earth Sciences, University of Bristol, Bristol, UK

**Keywords:** Induced seismicity, Shale gas, Hydraulic fracturing, North America

## Abstract

The Haynesville Shale in eastern Texas and western Louisiana has been one of the most productive shale gas plays in the USA. It is notable for being significantly over-pressured, a factor which has often been associated with an increased likelihood of hydraulic fracturing-induced seismicity (HF-IS) elsewhere. However, to date, only one case of HF-IS has been identified in the Haynesville play. Seismic monitoring across the play is relatively sparse, so it is possible that the absence of reported cases represents an absence of monitoring rather than an absence of cases. This study represents an investigation of HF-IS across the Haynesville play, primarily using data from the TexNet seismic monitoring array, which was installed in 2017. We use template matching to increase the population of detected earthquakes, increasing the number of detections by over 200% compared to the catalogs available from regional monitoring agencies. The resulting events can be clustered into several discrete sequences. We use an induced seismicity assessment framework to evaluate whether each sequence was induced and, if so, what industrial activity represents the most likely cause (both hydraulic fracturing and wastewater disposal operations take place within the footprint of the Haynesville play). We find three notable cases of HF-IS, straddling the region between Nacogdoches, San Augustine and Shelby Counties. Having identified these sequences, we examine whether any geological conditions may influence the occurrence of HF-IS. We identify increased formation depth, increased pore pressure gradients, and the thinning or absence of the underlying Louann Salt, which may otherwise serve as a hydraulic barrier between the Haynesville Shale and the basement, as factors that may account for the varying prevalence of HF-IS across the play.

**Supplementary Information:**

The online version contains supplementary material available at 10.1007/s10950-025-10296-x.

## Introduction

Hydraulic fracturing (HF) has caused cases of induced seismicity in different shale gas plays across northern America (Schultz et al. 2020; Verdon and Bommer 2021a). The rate at which hydraulic fracturing-induced seismicity (HF-IS) has occurred has varied significantly between different plays (Verdon and Rodríguez-Pradilla 2023). Some plays, such and the Montney, Duvernay, and Eagleford, have seen widespread and regular cases of HF-IS, while in others, such as the Marcellus, Barnett and Bakken, HF-IS has been rare or non-existent. These differences in HF-IS prevalence are primarily driven by the different geological settings, geomechanical properties and tectonic conditions pertaining to the different plays. However, the availability and quality of seismic monitoring has also influenced attempts to quantify rates of induced seismicity occurrence.

Induced seismicity hazard characterisation is generally based on past observations. Observed rates and magnitudes of induced seismicity are extrapolated forwards to characterise the hazard posed by future activities (e.g., Ghofrani and Atkinson 2016). This extrapolation process can be informed by variations in geological conditions between existing and potential future sites (Rodríguez-Pradilla and Verdon 2024). Recent studies have increasingly used compilations of induced seismicity cases to better understand the key factors and processes that govern the occurrence, rates and magnitudes of HF-IS (e.g., Pawley et al. 2018; Wozniakowska and Eaton 2020; Verdon and Rodríguez-Pradilla 2023). These efforts rely on consistent and accurate identification of cases of HF-IS: inaccurate or inadequate characterisation of past HF-IS cases can create biases in our estimation of induced seismicity hazard and thereby impact our ability to understand the factors that control HF-IS prevalence (Verdon and Bommer 2021b). The need to base our understanding of, and future estimation of, HF-IS hazard provides the motivation to review seismic monitoring datasets across shale gas plays. By doing so, we can identify previous cases of HF-IS that might otherwise have been missed.

The Haynesville Shale play straddles the border between Texas and Louisiana. To date, it has received relatively little attention with respect to HF-IS, with only a single reported instance (Walter et al. 2016). However, the broad-scale regional analysis presented by Verdon and Rodríguez-Pradilla (2023) identified additional potential cases of HF-IS within the Haynesville play that have not previously been studied. In this study, our objective is to analyse these cases in more detail to determine whether they represent as-yet undocumented cases of HF-IS.

Seismic monitoring coverage across the Haynesville play has been sparse. Limited coverage results in poor detection capability and location accuracy. In order to identify and correctly attribute potential cases of HF-IS, improvement of existing regional earthquake catalogs is often necessary. For example, detection of a larger number of earthquakes can help to identify when sequences started, accelerated and stopped. In turn, this information can be used to match sequences with specific hydraulic fracturing wells (e.g., did a sequence initiate before, during or after hydraulic fracturing took place in particular wells?). Likewise, accurate event locations are required to identify whether events are sufficiently close to specific wells for them to be considered a potential cause.

In this study, we used template matching to build a high-resolution catalog of earthquakes across the Haynesville play. We manually picked phase arrivals to invert for earthquake locations. We then compared the earthquake locations to hydraulic fracturing activities to identify potential cases of HF-IS. Having identified previously-undocumented cases of HF-IS in the Haynesville Shale, we examine whether there are any geological factors that might correlate with where HF-IS has occurred in the Haynesville play to date.

## The Haynesville Shale play: background, monitoring and induced seismicity

**Fig. 1:**
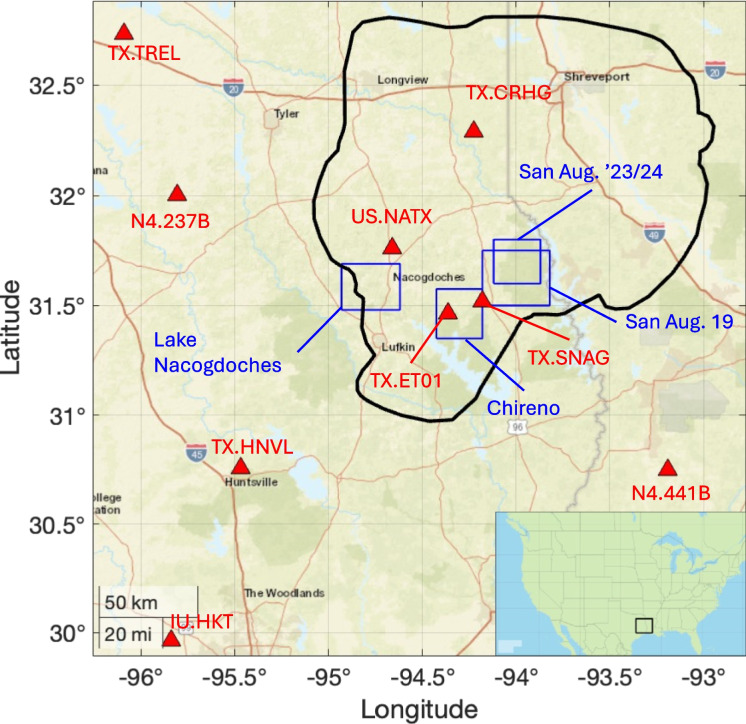
Map of our study area, showing the outline of the Haynesville Shale development area (black line), and the seismic stations used in our analysis (red triangles). The positions of our four detailed study areas (see Figs. 3–6) are marked with blue squares. Shale play boundaries are from EIA (2016). The inset map (lower right) shows the position of the study area with respect to the USA

The Haynesville Shale is a Jurassic-age formation that straddles east Texas, northwest Louisiana and southwest Arkansas. It is prospective for gas production within a region extending over 170 × 170 km centred on the Louisiana-Texas border (Fig. 1). Permeability is in the nanodarcy range (Wang et al. 2013a), such that high volume hydraulic fracturing is used to achieve commercial production rates. Typical HF volumes are of the order of tens of thousands of cubic metres per well (Nicot and Scanlon 2012). The Haynesville Shale has been one of the most productive shale plays in the USA: production initiated in 2009 and initially peaked in 2012 at over 6 billion cubic feet per day (bcf/d), fell back over the mid 2010 s, before increasing again from 2018 onwards, reaching a new peak of over 14 bcf/d in 2023 (EIA 2024).

The Timpson induced seismicity sequence is found within the Haynesville play footprint. This sequence has been extensively studied (Frohlich et al. 2014; Fan et al. 2016; Wang et al. 2020), and found to be associated with wastewater disposal (WWD) activities. The Timpson sequence initiated in 2008 and peaked with an M_W_ 4.8 event in May 2012. Seismicity has continued in this sequence, albeit at lower levels, until at least 2020 (Watkins et al. 2023). However, whereas the Timpson WWD-induced seismicity sequence has received extensive study, hydraulic fracturing activities in the Haynesville Shale have received little attention with respect to induced seismicity. Only one confirmed case of HF-IS in the Haynesville play has been identified to date: the August – October 2011 Bienville Parish sequence in northwestern Louisiana, which reached a maximum magnitude of M_L_ 1.9 (Walter et al. 2016).

### Seismic monitoring

Figure 1 shows the seismic monitoring stations from which data is available for this study. The only seismic station to have remained in place throughout the time that the Haynesville has been under development is the US National Seismic Network station US.NATX. From 2010 to 2012, the USArray Transportable Array (TA) experiment traversed this area. Walter et al. (2016) used data from this array to identify clusters of earthquakes within the Haynesville play that were likely associated with HF and WWD. Two TA sites were converted into permanent stations from 2012 (N4.237B and N4.441B), though both of these sites are more than 50 km from the Haynesville play. After the 2012/05/17 MW 4.8 Timpson mainshock, additional temporary monitoring stations were installed in the immediate area during 2012–2013.

From 2017, the TexNet array (Savvaidis et al. 2019) has provided coverage for the eastern portion of the play. Two stations, TX.SNAG and TX.CRHG, are available within the Haynesville play area from 2017 onwards, with a further station, TX.ET01, being available from early 2019 (see Fig. 1). A further two stations, TX.TREL and TX.HNVL, are available from 2017 within approximately 100–200 km of the Haynesville play area. The Global Seismograph Network station IU.HKT is located roughly 200 km to the southwest. A network of seismometers was installed to monitor induced seismicity across western Louisiana between 2019 to 2022 (Kraus et al. 2021), but data from this network is not publicly available at present.

### Earthquake catalogs

**Fig. 2:**
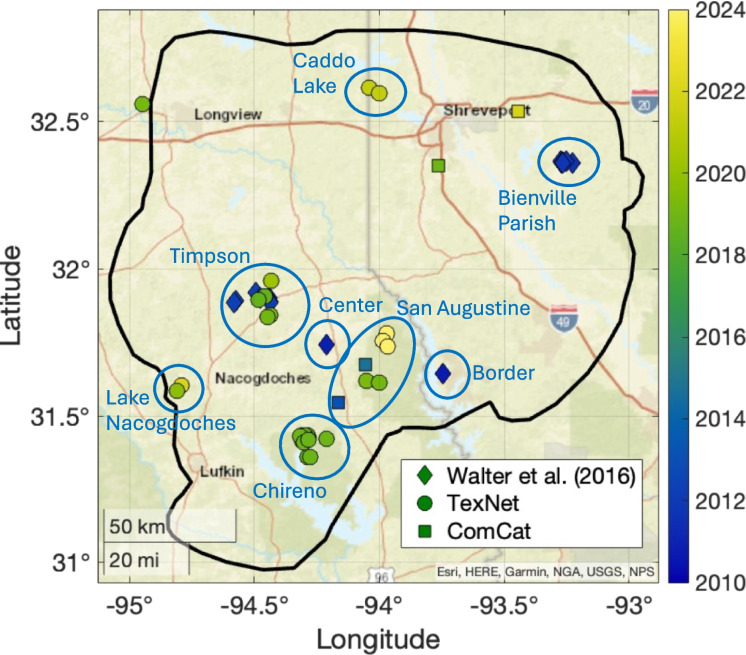
Map of earthquakes from existing catalogs within the Haynesville Shale play area. Various clusters defined by Walter et al. (2016) and in this study are highlighted

Figure 2 shows earthquakes cataloged in the Haynesville play area to date. These events are drawn from the catalog produced using USArray TA data by Walter et al. (2016), which runs from 2010–2012, and from the TexNet catalog, which runs from 2017 to present. To fill the gap from 2012–2017, we used events drawn from the USGS ComCat catalog, recognising that this catalog likely has a much poorer detection capability and location accuracy given the absence of monitoring stations in the area during this time.

The earthquake clusters defined by Walter et al. (2016) are shown in Fig. 2, including Timpson (caused by WWD), Bienville Parish (caused by HF), and the Center and Border clusters, the causes of which were not established by Walter et al. (2016) (HF and WWD activities were both taking place nearby at the time). The catalog events are listed in our Supplementary Materials Table S2.

Clusters of events at Chireno, San Augustine, Lake Nacogdoches, and Caddo Lake are seen in the TexNet catalog from 2017 onwards. We study earthquakes occurring from 2017 until the end of 2023. From 2017, the additional coverage provided by the TexNet stations represents a significant improvement over previous monitoring (apart from the period from 2010–2012 when coverage was provided by the USArray TA, but this data has already been analysed by Walter et al. 2016).

We do not examine the Timpson cluster, since this has already been the subject of extensive study (Frohlich et al. 2014; Fan et al. 2016; Wang et al. 2020), and most of the seismicity in that sequence occurred prior to 2017. These studies robustly established that the Timpson events were caused by WWD. We do not examine the Caddo Lake cluster because this sequence occurs in close proximity to stations in the Louisiana induced seismicity monitoring network, the data from which is not publicly available. However, in our Supplementary Material we briefly examine whether the Caddo Lake sequence is likely to be a case of HF-IS based on the TexNet catalog information.

For induced seismicity attribution assessments, knowledge of the background or baseline “natural” earthquake rates is often required (e.g., Davis and Frohlich 1993; Verdon et al. 2019). However, historic seismicity monitoring in this area has been of limited quality. Frohlich and Davis (2003) provides the most comprehensive documentation of historic earthquakes in Texas that we are aware of. This study shows that rates of seismicity in the area have been extremely low. Based on USGS ComCat data, magnitudes of completeness in the area prior to 1980 appear to be at least M 3.0, and as high as M 2.5 up to at least 2000, and therefore of limited use for this study where the majority of observed events are smaller than M 2.5. Moreover, any assessment of baseline natural seismicity rates for the area is further complicated by the fact that many historic earthquakes in Texas may also have been induced (Frohlich et al. 2016): oil and gas activities in Texas long precede any kind of systematic seismic observation.

### Hydraulic fracturing and wastewater disposal well datasets

As of October 2011 and February 2012 respectively, operators in Texas and Louisiana have been required to report HF well injection volumes and start/end times to the FracFocus registry (http://www.FracFocus.org). Some wells were voluntarily reported to FracFocus before these dates. The FracFocus database forms our primary source for HF well information in this study. The FracFocus database is now widely used to assess the environmental impacts of hydraulic fracturing (Dundon et al. 2015).

The FracFocus database gives a single location for each well. Maximum well depths are also reported, but not the specific formation being targeted. Most HF wells in the Haynesville include long lateral sections extending over hundreds or thousands of metres. Both the wellhead position and the well “toe” are required to fully delineate the areas in which HF has taken place. HF well data is also available via the Texas Railroad Commission (RRC) and the Louisiana Department of Energy and Natural Resources (DNR). The RRC and DNR HF well datasets include positions for the wellhead and the downhole toe of the well. From inspection, well locations in the FracFocus database sometimes correspond to well surface locations, and sometimes to the downhole toe of the well. For wells prior to 2011/2012 that are not reported to FracFocus, well license information in the RRC and DNR databases can be used to give some indication of when operations may have taken place, but specific start/end times and injection volumes are not available. WWD data for Texas is available via the RRC Underground Injection Control (UIC) database, which includes well locations, monthly injection rates, and the top and bottom of the disposal interval, but not the name of the formation(s) being targeted. The Louisiana DNR provides location maps of WWD wells, but we were not able to identify any data for injection volumes or timings.

## **M**ethods

### Earthquake detection

We used template matching (Gibbons and Ringdal 2006) to identify additional earthquakes that were not reported in the TexNet catalog. For each cluster (as defined in Fig. 2) we used all the TexNet catalog events in that cluster as templates. The specific templates used, and time periods analysed by template matching, are listed in Table S3. We generally searched time windows of 2–3 months before and after any template events.

We based our template matching on the nearest available station for each cluster: for the Chireno cluster we used TX.SNAG (TX.ET01 had not been installed when this cluster initiated); for the San Augustine clusters we used TX.SNAG and TX.ET01, and for the Lake Nacogdoches cluster we used TX.ET01. To generate the templates, we first high pass filtered the data at 1 Hz. We then manually selected data windows starting just before the P-wave onset and ending as the S-wave coda abates. The resulting templates typically have a length of around 20 to 30 s.

We calculated normalised cross-correlation coefficients, *NCC*, between every template in the cluster and the continuous seismic traces (which were also filtered with a 1 Hz high pass). We used a relatively conservative cross-correlation threshold of max(*NCC*) ≥ 0.15 to identify candidate events, where a detection takes place if the maximum cross-correlation value exceeds this threshold for any single template event within the template catalog. Each event candidate was then manually inspected to ensure that it represented a true earthquake detection. We found that the threshold of max(*NCC*) ≥ 0.15 provided a good balance between detecting a high number of events while avoiding false positives.

For the events identified by template matching, we found some cases where seismic arrivals could be observed on multiple stations within the network. These events were taken forward for event location, as described below. For many of the events identified by template matching, signal strength was low such that seismic arrivals could not be identified on any other stations in the network.

### Earthquake locations and magnitudes

Where P- and/or S-wave arrivals could be identified on at least 4 stations, we performed a manual event location. The catalog of “locatable” events included events already identified in the regional catalogs described above, as well as events newly identified by the template matching. P- and S-wave arrival times were picked manually and inverted for the best-fit location that minimised the least-squares differential between modelled and observed arrival times. P- and S-wave travel times were modelled using an Eikonal solver (Podvin and Lecomte 1991), using the 1D layered velocity model for east Texas published by Borgfeldt (2017). We used the Neighbourhood Algorithm (Sambridge 1999) to search for the best-fitting event location that minimises travel time residuals. We performed an iterative procedure to estimate location uncertainties, whereby observed pick times were perturbed within prescribed uncertainty windows, with the resulting distribution of locations defining the location uncertainty.

For the “unlocated” events with visible seismic arrivals on fewer than 3 stations, we used the cross-correlation coefficients from the template matching analysis as a guide to an approximate or indicative event location. We did so on the basis that a high CC value implies that the test event location is near to that of the template (Gao and Kao 2020). Many of the newly identified “test” events had high *NCC* values for multiple templates. Where this was the case, we produced an estimate of the test event location as a weighted average of the locations of the template events with *NCC* values that exceeded our detection threshold:

1 $${\overrightarrow{x}}_{test}=\frac{\sum_{i=1}^{n}{CC}_{max}(i){\overrightarrow{x}}_{temp}(i)}{\sum_{i=1}^{n}CC(i)}$$

where ${\overrightarrow{x}}_{test}$ is the location (in Lat/Lon and depth) of the test event to be estimated, ${\overrightarrow{x}}_{temp}$ is the location of each of the *n* template events for which max(*NCC*) ≥ 0.15, and *CC*_*max*_ is the maximum *NCC* value between the test event and each of the template events. Where a test event has max(*NCC*) ≥ 0.15 for only one template event, the test event is treated as occurring at the same position as that template event. We note that this approach is far from optimal, but it does nevertheless provide a rough sense of where within each cluster the unlocated events are likely to have originated.

We used the scale published by Kavoura et al. (2020) to compute local magnitude values, M_L_, for all events. For events with seismic arrivals visible on multiple stations we adopted the mean M_L_ value between stations.

A full list of earthquakes identified in this study is provided in the Supplementary Materials, including events for which accurate locations were obtained, and events identified by template matching for which locations were estimated based on template similarity using Eq. 1.

### Induced seismicity assessment

Determining if a sequence of events is induced, and if so, by what activity, can be challenging. This assessment is made more challenging still when different activities, such as HF and WWD, are taking place in the same area (e.g., Yoon et al. 2017). Seismic waveforms generated by induced earthquakes have the same character as those generated by natural earthquakes. Attribution of induced seismicity must instead be done by comparing the spatial and temporal evolution of the seismicity with the timings and positions of industrial activities. High spatial and temporal correlation between earthquakes and industrial activities, at a level that would be unlikely to occur naturally by chance, is typically taken as evidence for earthquakes being induced.

These insights have been used to develop frameworks within which potential induced seismicity causation can be assessed. These frameworks typically pose a series of questions pertaining to the locations of events relative to the proposed industrial cause, the timings of events relative to the timings of industrial activities, and other questions as to whether the proposed activities could have created sufficient perturbations at the locations of the earthquakes. The earliest such framework is that of Davis and Frohlich (1993). Verdon et al. (2019) produced an updated framework which incorporated a more nuanced approach to handling incomplete datasets and uncertainties. We adopted the Verdon et al. (2019) framework to assess whether the identified clusters of events were induced and, if so, by which activities.

The quality of evidence used in an induced seismicity assessment is quantified in the Verdon et al. (2019) framework by the Evidence Strength Ratio (ESR). The higher the ESR score, the better the available evidence used to make the induced seismicity assessment. The outcome of the Verdon et al. (2019) framework is quantified by the Induced Assessment Ratio (IAR), with a negative IAR implying that activity under consideration is not likely to be the cause of the seismicity, while a positive IAR implies that the activity under consideration is the likely cause. A high IAR score implies a more certain conclusion: IAR scores within 10–20 percentage points of 0 (whether positive or negative) imply that the induced seismicity attribution is ambiguous or unclear.

Where sufficient data is available, the Verdon et al. (2019) method can be informed by statistical observations that quantitatively evaluate the spatial and temporal distributions of earthquakes relative to industrial operations. However, where data availability is limited, for example where earthquake populations are small, or where operational data (e.g., injection rates) are not publicly available, then the Verdon et al. (2019) scheme can be informed by more qualitative judgements. The ESR score in the Verdon et al. (2019) scheme plays key a role in characterising the strength of the evidence used to make these judgements. We note that purely quantitative schemes for induced seismicity assessment have also been developed (e.g., Dahm et al. 2013; Llenos and Michael 2013; Kothari et al. 2020). In this study, the low numbers of earthquakes observed and the limited availability of industrial data do not permit the use of such methods. The Verdon et al. (2019) scheme, where the strength of evidence can be directly quantified, is therefore ideally suited for this application.

## **R**esults

### Chireno cluster

**Fig. 3:**
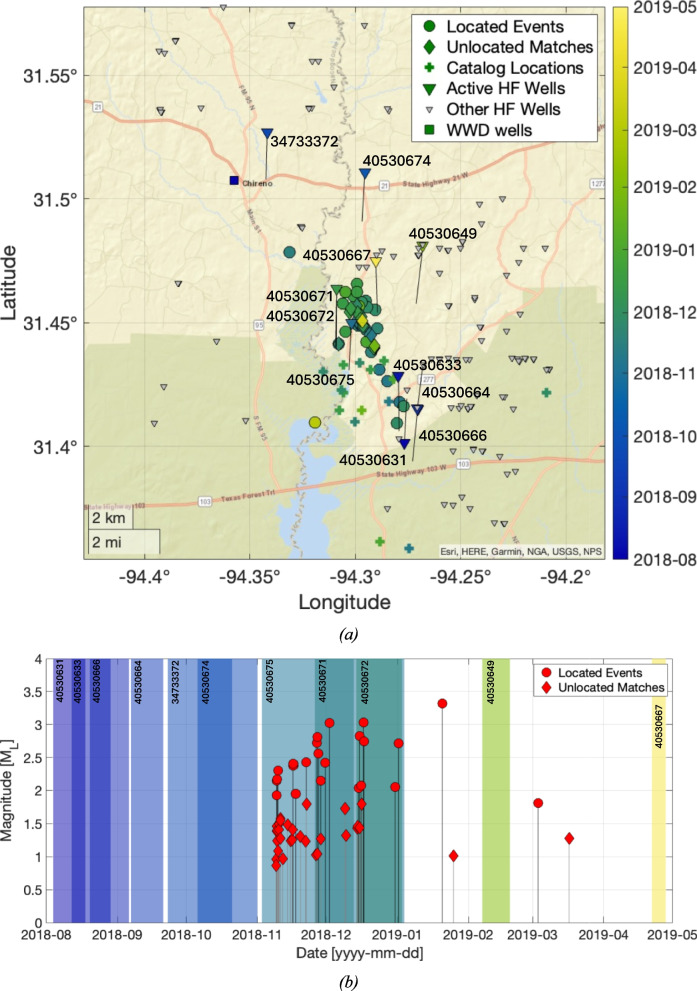
Map (**a**) and timeline (**b**) of earthquakes and hydraulic fracturing operations for the Chireno cluster between November 2018 to April 2019. In (**a**), coloured circles show the located events (coloured by occurrence time), and diamonds show the positions of the unlocated events estimated by Eq. 1. The + symbols show event locations from the TexNet catalog. Large, coloured triangles show surface wellhead positions for wells in which HF operations took place during the time of interest (coloured by the start date of stimulation), with black lines showing the track of the wells in the subsurface, from the wellhead to the well toe. The surface wellhead positions for other HF wells that were not active during this time are shown as small grey triangles. Active WWD wells are shown as squares. In (**b**), the located events are shown as red circles, and the unlocated template matches are shown as red diamonds. The coloured patches show the periods during which HF operations were taking place in each well (labelled with API numbers)

The Chireno sequence took place between November 2018 to April 2019 and contained a total of 54 detected events – the highest number of events in any of our re-analysed sequences. Magnitudes in this cluster ranged from M_L_ 0.9 to M_L_ 3.3. We have not attempted to assess the magnitude of completeness, as interrogation of the Gutenberg-Richter distribution would be unlikely to produce statistically significant results given the small number of detected events (e.g., Roberts et al. 2015). A map and timeline for the 2018/2019 Chireno sequence is shown in Fig. 3. The events fall along a trend extending NNW-SSE over a distance of about 5 km. Within this cluster, events typically have uncertainties in the N-S axis of around 2–3 km, and uncertainties in the E-W axis of around 1–1.5 km. Hence, the apparent elongation of the cluster in a N-S direction may to a degree reflect the uncertainties in event locations. Depth uncertainties for these events range from 2–3 km. All the events are located at depths shallower than 6 km. HF operations in the Haynesville in this area take place at depths of around 4,000 m.

Our re-locations place the events slightly to the north of the TexNet catalog locations. The timing and positioning of the events overlaps with hydraulic fracturing operations in wells 40530675, 40530671, and 40530672 (well API numbers). Well 40530675 began HF operations on 2018/11/03. The first events within the sequence were observed on 2018/11/09. Wells 40530671 and 40530672 were drilled from the same pad in very close proximity to each other – HF operations in these wells initiated on 2018/11/26 and 2018/12/14 respectively. A single WWD well (well 34733181) is located approximately 7 km to the northwest. Disposal in this well started in December 2011. Disposal in this well took place at approximately 1,600 m depth, more than 2,000 m shallower than the hydraulic fracturing activities.

Our full assessment of induced seismicity causation via the Verdon et al. (2019) framework is provided in the Supplementary Materials. We find a very high likelihood that the Chireno events were induced by the hydraulic fracturing activities in the three identified wells. The largest event in this sequence reached M_L_ 3.3, occurring on 2019/01/20. The last HF operations in the three identified wells had finished on 2019/01/04 (well 40530675). The time delay of 14 days between the end of HF operations and the largest event is towards the longer end of the observed range for trailing events for HF-IS sequences (Verdon and Bommer 2021a). After the M_L_ 3.3 event, the occurrence of seismicity rapidly decreased, with only three further events detected.

### San Augustine 2019 cluster

Two events were recorded within the San Augustine area, on 2013/02/03 and 2014/10/03, in the USGS ComCat catalog (see Fig. 2). Given the lack of seismic coverage in the area at the time, these events do not form part of our analysis. Two further bursts of events within the San Augustine area are identified by our analysis, occurring in 2019 and 2023/24.

**Fig. 4:**
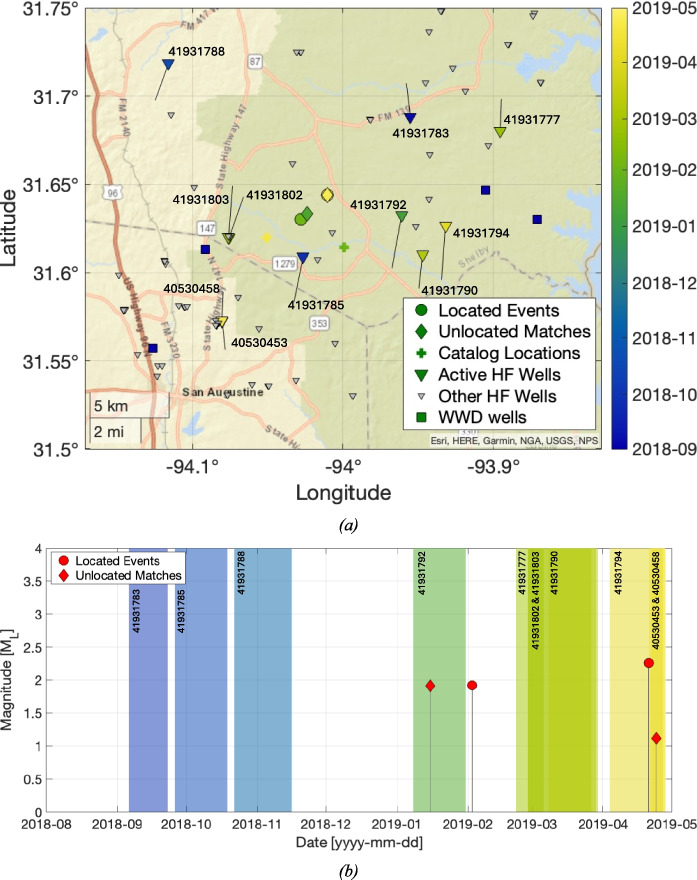
Map (**a**) and timeline (**b**) of earthquakes and hydraulic fracturing operations for the 2019 San Augustine cluster. Figure formats are as per Fig. 3

A total of four events were identified in the 2019 San Augustine sequence. Magnitudes in this cluster ranged from M_L_ 1.1 to M_L_ 2.3. A map and timeline for these events is shown in Fig. 4. The events are all located within roughly 2 km of each other, and at depths of less than 2 km. Uncertainties for these events are as much as 4 km East–West, 2 km North–South, and over 3 km in depth. The TexNet catalog locations for the two locatable events straddle our locations, being approximately 2 km to the southeast and southwest.

Several HF wells were active in the area when these events occurred, as well as four active WWD wells (wells 41930529, 41931048, 40530213, 40530468). As with the Chireno cluster, hydraulic fracturing in the Haynesville occurs at depths of around 4,000 m, while the deepest WWD reached approximately 2,100 m depth. The WWD wells began injecting between 2005 and 2012. The events can be further divided into two sub-clusters, each of which coincided with a period when a specific HF well was active. The first two events occurred in Jan/Feb 2019, when well 41931792 was active. The second two events occurred in late April 2019, when well 41931794 was active.

Our full assessment of induced seismicity causation via the Verdon et al. (2019) framework is provided in the Supplementary Materials. Our assessment produces moderate positive IAR scores for both HF and for WWD operations, indicating that the events are likely induced but that there is ambiguity as to the causation of these events between HF and WWD activities. The IAR score for HF is higher, implying that this is the more likely cause, but the role of WWD cannot be ruled out. Given that WWD has been ongoing in the area for a significant period of time, we might expect a longer ongoing sequence of seismicity were this the main driving factor. In contrast, the 2019 San Augustine cluster consists of two brief bursts, both of which coincide with HF operations in nearby wells. However, the location uncertainties for this cluster are relatively high, and it is possible that the events may be too far from the HF wells for them to represent a plausible cause.

### San Augustine cluster 2023/24

**Fig. 5:**
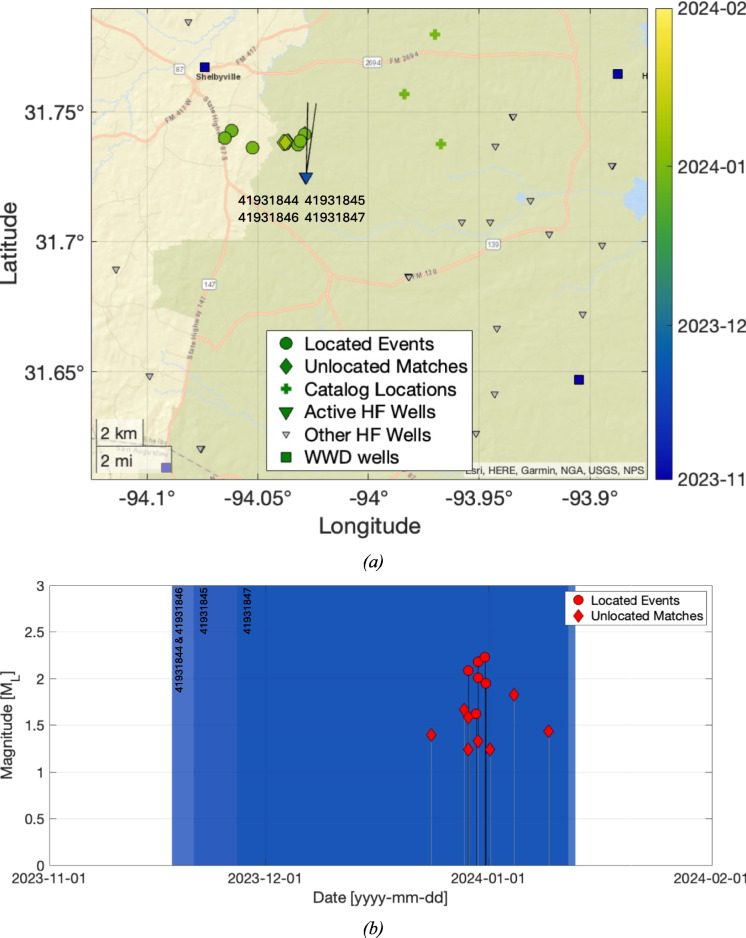
Map (**a**) and timeline (**b**) of earthquakes and hydraulic fracturing operations for the 2023/24 San Augustine cluster. Figure formats are as per Fig. 3

The 2023/24 sequence within the San Augustine area is located to the north of the 2019 San Augustine sequence. A total of 14 events were identified in this sequence: a map and timeline for these events is shown in Fig. 5. The events are all located within roughly 4 km of each other, extending westwards from the horizontal laterals of four HF wells (wells 41931844, 41931845, 41931846, and 41931847). HF operations were ongoing in these wells at the time that the events occurred. Magnitudes in this cluster ranged from M_L_ 1.2 to M_L_ 2.3.

The events are located at depths of between 2–7 km. Uncertainties for these events are roughly 2–4 km East–West, 1–3 km North–South, and over 6 km in depth. Hence, the apparent elongation of the cluster in an E-W direction may simply reflect the uncertainties in event locations. The TexNet catalog locations for these events were between 5–10 km to the east of our locations, placing them significantly further from the identified active HF wells.

Three WWD wells are also present in the area (wells 41930466, 41931048, and 40530468), however the nearest of these (well 41930466) ceased injection in 2006, while well 40530468 ceased injection in 2018. WWD depths ranged from 1,000–2100 m.

Our full assessment of induced seismicity causation via the Verdon et al. (2019) framework is provided in the Supplementary Materials. We find a very high likelihood that the 2023/24 San Augustine events were induced by the HF operations in the four identified HF wells: the event locations directly coincide with the position of the wells, and the sequence began and ended when hydraulic fracturing was ongoing in these wells. In contrast, the only active WWD more than 16 km from the events. As described above, for long-term, ongoing WWD activities we might expect to see longer-duration induced seismicity sequences if WWD were the primary driving factor.

### Lake Nacogdoches cluster

Two events were recorded towards the eastern edge of the Haynesville play, near to Lake Nacogdoches. This included one of the largest events in the TexNet catalog for the Haynesville play, with M_L_ 3.2, with the other event having a magnitude of M_L_ 2.8. The first event occurred in October 2019, and the second in December 2021. Both of these events are identified in the TexNet catalog. Our template matching search did not identify any additional events in this cluster, implying that the two recorded events are isolated instances, rather than occurring within more populous sequences, as was the case for the other sequences discussed above.

**Fig. 6:**
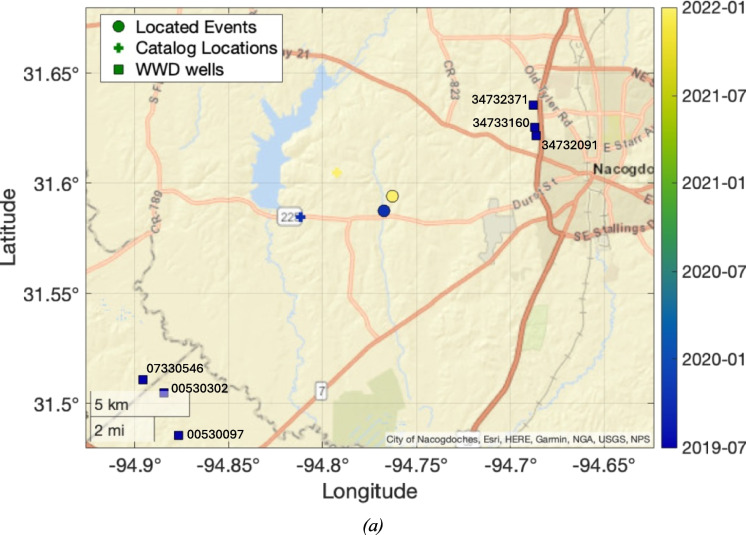
Map (a) of earthquakes and WWD operations for the Lake Nacogdoches cluster. Figure formats are as per Fig. 3

Figure 6 shows a map of the events. There are no HF wells in the vicinity of these events. However, six WWD wells are nearby, the closest of which are within 8 km. The deepest WWD reached 2,800 m, but most of the WWD wells target depths between 1,700–2,000 m. Our full assessment of induced seismicity causation via the Verdon et al. (2019) framework is provided in the Supplementary Materials. Our outcome is ambiguous for these events, with a low IAR score indicating that the events could be induced or natural. The events are sufficiently close to the WWD wells for them to be considered as a potential cause. However, the rate of seismicity within this cluster, consisting of only two events over a period of more than two years, does not seem out of place for background rates of seismicity in the region (Frohlich and Davis 2003).

## **D**iscussion

Our analysis has identified several cases of HF-IS in the Haynesville Shale. These cases are predominantly found along the southern edge of the play. It is therefore worth examining the extent to which different geological factors may control the prevalence of HF-IS across the play.

Induced seismicity occurs when fluid injection causes perturbations on pre-existing tectonic faults. These perturbations may be caused by stress transfer through the rock frame (e.g., Kettlety et al. 2020; Igonin et al. 2022) or by direct hydraulic connection into the fault (e.g., Igonin et al. 2021): what these mechanisms share is the need for a pre-existing fault that is close to shear failure conditions in the in situ stress field (often expressed in terms of the Mohr–Coulomb failure envelope). Hence, from a theoretical consideration, geological conditions that increase the abundance of so-called critically stressed faults will serve to increase the likelihood of HF-IS. These conditions might include an increased abundance of faulting, the presence of faults with optimal orientations in the in situ stress field, higher shear stresses, and increased pore pressures.

Verdon and Rodríguez-Pradilla (2023) identified several factors that jointly controlled the prevalence of HF-IS between different shale gas plays in North America. These included the pore pressure gradient, with elevated pore pressures serving to reduce effective normal stresses and unclamp faults, and the in situ stress field classification, as quantified using the Simpson (1997) *A*_*ϕ*_ parameter, with reverse-faulting conditions (*A*_*ϕ*_ values > 1.5) increasing the in situ shear stresses on critically-stressed faults. The Haynesville Shale is among the most over-pressured of any shale play in North America, with pore pressure gradients as high as 20 kPa/m. However, it sits in a region with extensional to strike-slip stress conditions (*A*_*ϕ*_ ≈ 1).

Within individual plays, various risk factors for HF-IS have been observed, including elevated pore pressures (Eaton and Schultz 2018), proximity to basement (Skoumal et al. 2018), formation depth (Ries et al. 2020), and proximity to mapped faults (McKeighan et al. 2022) or geological proxies thereof (e.g., Schultz et al. 2016).

**Fig. 7:**
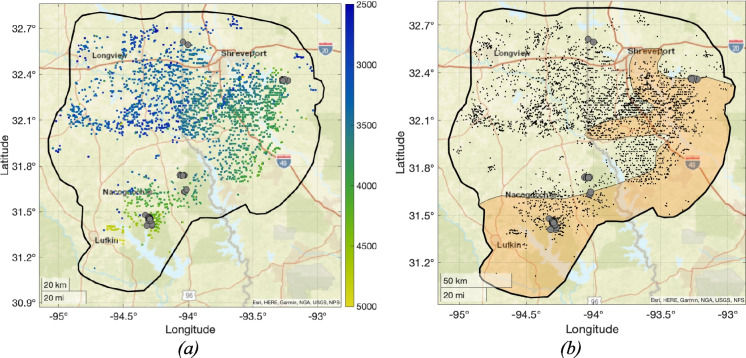
Maps of pressure and well depth across the Haynesville play. In (**a**), we show the reported TVD for each well (metres below sea level). In (**b**) the orange polygon shows the area mapped by Wang et al. (2013b) in which the pressure gradient in the Haynesville exceeds 0.9 psi/ft (20.36 kPa/m). The grey dots show the locations of earthquakes that we have identified as likely to represent cases of HF-IS

Figure 7a shows a map of well depths (total vertical depth below sea, TVD) within the Haynesville play. We note that some of the wells to the northwest of the area target the shallower Cotton Valley Formation. There is a clear trend of increasing formation depth to the south and southeast. Wang et al. (2013b) mapped pore pressure gradients in the Haynesville Formation, identifying that pore pressure gradients were highest (> 0.9 psi/ft, or 20 kPa/m) in the south and east of the formation (see Fig. 3 of that paper). The zone of highest pore pressure gradients mapped by Wang et al. (2013b) is shown in Fig. 7b.

Comparing the formation depth and pressure gradients with the locations of HF-IS identified in this study, we find that most of the HF-IS cases are found where the Haynesville Formation is at greater depth, and within or adjacent to areas with higher pore pressure gradients. The implication that increasing formation depth and increasing pore pressure gradient are risk factors for HF-IS in the Haynesville is consistent with observations elsewhere (e.g., Eaton and Schultz 2018; Ries et al. 2020). Increased formation depth can drive increased induced seismicity risk for a number of reasons, including the potential for higher pore pressure gradients, and increased proximity to basement.

Numerous studies have noted the importance of proximity to, and hydraulic connections into, basement rocks (e.g., Skoumal et al. 2018; Pawley et al. 2018; Hincks et al. 2018). Basement rocks are typically stiffer, and therefore able to support higher shear stresses; and they are older, and therefore likely to contain a higher density of faulting. Skoumal et al. (2018) assessed the occurrence of HF-IS in the Appalachian Basin, finding that HF-IS was more common during stimulation of the deeper Utica Formation, which lies close to the basement, whereas HF-IS was very rare during stimulation of the overlying Marcellus Formation. The Marcellus is isolated from the basement by the Salina Group evaporites, and Skoumal et al. (2018) argued that the presence of the Salina Group provides a hydraulic and geomechanical barrier between the Marcellus and basement rocks, which may account for the absence of HF-IS in the Marcellus.

A similar situation pertains in the Haynesville play, where the Haynesville Formation is underlain by the Louann evaporite deposits, which could provide a hydraulic barrier to the basement. However, the Louann Formation varies in thickness as it runs below the Haynesville. The southern portion of the play is influenced by the Sabine Island uplift, a palaeogeographic high that formed during the Triassic rifting of the northern Gulf of Mexico Basin (Adams 2009). The presence of the Sabine Island uplift influenced deposition during the Jurassic, such that the Louann Salt is significantly thinned or absent in the Shelby Trough (the area south of the Strickland High, encompassing Shelby, San Augustine and Sabine Counties). This thinning of the Louann Formation and other strata underlying the Haynesville is visible in reflection seismic surveys (see Fig. 3 of Cicero and Steinhoff 2013) and well correlations (see Fig. 10 of Hammes et al. 2011). Within the Haynesville play, the Shelby Trough has the highest abundance of HF-IS, as it contains the Chireno and San Augustine clusters. The implication that the thinning of the Louann Salt around areas of palaeogeographic uplift, which could facilitate hydraulic connections from the Haynesville Formation into the underlying basement, is a risk factor for HF-IS is again consistent with HF-IS risk factors observed elsewhere (e.g., Skoumal et al. 2018). We have not compared the positions of HF-IS cases against mapped faults, as we are not aware of any publicly available fault databases for the region. Clearly, a comparison between mapped faults and HF-IS cases would be a worthwhile exercise for future research.

It should be noted that the Caddo Lake cluster, which also likely represents a case of HF-IS, occurs towards the north of the play (straddling the state border between Harrison County and Caddo Parish) in an area where the Louann Salt is present, where pore pressure gradients are lower (relative to the south and eastern areas – they are still high relative to many plays elsewhere), and where the Haynesville is shallower. Also, the occurrence of HF-IS appears to be highly spatially variable: there are many HF wells near to those that caused the Chireno and San Augustine clusters (see Figs. 3, 4 and 5) that did not produce any reported HF-IS. The overall rate of HF-IS for the Haynesville remains low (Verdon and Rodríguez-Pradilla 2023), and even in areas with elevated risk only an unlucky few wells have experienced HF-IS.

These observations demonstrate the need to treat HF-IS risk factors in a stochastic or probabilistic manner (e.g., Gupta and Baker 2019; Teng and Baker 2020; Rodríguez-Pradilla and Verdon 2024). The presence or absence of various risk factors may indicate an increased or decreased likelihood of HF-IS occurrence for a given area. However, the presence of risk factors does not mean that a given well is inevitably destined to experience HF-IS (as witnessed by the many wells that did not experience HF-IS, despite being very close to wells that did), and the relative absence of risk factors cannot be used to entirely preclude the possibility of HF-IS occurrence for a given well (as witnessed by the Caddo Lake sequence, which occurred despite an apparent absence of the identified risk factors).

## **C**onclusions

We have conducted an appraisal of HF-IS in the Haynesville Shale of eastern Texas and Louisiana. This formation is generally thought to have a low prevalence of induced seismicity, although seismic monitoring in the region has been relatively sparse. We used template matching to identify earthquakes that were not detected by existing regional catalogs for the area. From an original catalog of 23 total templates, we were able to detect an additional 51 previously uncataloged events. We performed manual re-locations for each detected event where phase arrivals could be identified on a sufficient number of stations. We estimated indicative positions for the remaining events based on the similarity of their waveforms to the template events.

From the resulting earthquake catalog, we clustered the events into four distinct sequences. We compared each event sequence to nearby HF and WWD operations and used the Verdon et al. (2019) framework to assess whether each sequence was induced or natural, and if induced, what activity was the likely cause. We found that the Chireno and San Augustine 2019 and 2023 sequences were likely caused by HF operations in the Haynesville Shale. Causation for the Lake Nacogdoches sequence was ambiguous: there were no nearby HF operations, but it is possible that the events were natural, or that they were caused by WWD operations.

Having identified these cases of HF-IS, we compare their locations with the regional geological conditions across the Haynesville Shale. By doing so, we aim to better understand the different geological factors that may serve to promote the occurrence of HF-IS. HF-IS is most abundant across the southern and eastern portions of the play. These areas correspond to areas where the Haynesville Shale is deeper, with higher pore pressure gradients. These areas also correspond to areas where the Louann Salt, which might otherwise represent a hydraulic barrier between the Haynesville and the underlying basement, is absent or significantly thinned. The risk factors for HF-IS identified in this study: operations in deeper formations, higher pore pressure gradients, and with hydraulic connections to basement rocks, are consistent with previous findings in other shale plays.

## Supplementary Information

Below is the link to the electronic supplementary material.

Supplementary file1 (PDF 1.25 MB)

## Data Availability

The seismic waveforms analysed in this study were sourced from IRIS. The earthquake catalogues were sourced from USGS ComCat (https://earthquake.usgs.gov/data/comcat/), TexNet (https://www.beg.utexas.edu/texnet-cisr/texnet/earthquake-catalog) and material published in Walter et al. (2016). Well data were sourced from FracFocus (https://www.fracfocus.org), the Texas Railroad Commission (https://gis.rrc.texas.gov/GISViewer/), and the Louisiana Department of Natural Resources (https://www.sonris.com).
