# Supplementary material for: Investigation of hydraulic fracturing-induced seismicity in the Haynesville Shale
Source: J Seismol. 2025 May 19;29(3):625–41. doi: 10.1007/s10950-025-10296-x (PMC12271280; doi:10.1007/s10950-025-10296-x)
Supplement: Supplementary file 1 — Supplementary file1 (PDF 1.25 MB) [file 10950_2025_10296_MOESM1_ESM.pdf]

Investigation of hydraulic-fracturing induced seismicity in the Haynesville Shale

James P. Verdon, Alexander D.G. Harris

Supplementary Material

This supplementary material contains the following material:

Table S1: List of seismic monitoring stations used in our analysis

Table S2: List of earthquakes catalogued by Walter et al. (2016), TexNet, and ComCat.

Table S3: List of templates used for each cluster, and time periods analysed by template matching.

Table S4: List of all events detected and relocated in this study

Table S5: List of all events detected but with insufficient number of phases to be located

Table S6: Induced seismicity assessment for the Chireno 2018/19 cluster

Table S7: Induced seismicity assessment for the San Augustine 2019 cluster

Table S8: Induced seismicity assessment for the San Augustine 2023/24 cluster

Table S9: Induced seismicity assessment for the Lake Nacogdoches cluster

Text Section S10, Figure S11 and Table S12: Discussion and analysis of the Caddo Lake cluster

Table S1: List of seismic stations used in this study

Station	Network	Lat [°]	Lon [°]	Elevation [m]	Sampling rate [Hz]	Recording from
CRHG	TX	32.289	-94.225	95	100	2017/09/28
HNVL	TX	30.756	-95.467	87	200	2017/04/06
SNAG	TX	31.518	-94.179	84	200	2017/08/07
TREL	TX	37.730	-96.089	174	200	2017/08/07
237B	N4	32.002	-95.808	116	100	2014/02/05
441B	N4	30.750	-93.190	33	100	2014/01/15
HKT	IU	29.965	-95.838	69	40	1995/07/11
NATX	US	31.760	-94.661	168	40	2004/05/12
ET01	TX	31.465	-94.356	127	100	2019/01/08

Table S2: List of catalogued events in the region. W2016 = sourced from Walter et al. (2016); ComCat = the USGS comprehensive catalog, TexNet = the TexNet catalog.

Date	Time	Magnitude	Lat [°]	Lon [°]	Depth [m]	Source
22/04/2010	03:00:40	0.8	31.884	-94.436	300	W2016
17/06/2010	20:05:26	1.2	31.884	-94.435	300	W2016
20/06/2010	06:17:16	1.1	31.744	-94.21	5100	W2016
20/06/2010	09:23:27	0.9	31.745	-94.209	5100	W2016
21/06/2010	04:42:29	0.9	31.744	-94.209	5100	W2016
21/06/2010	07:36:36	0.8	31.744	-94.21	5100	W2016
27/06/2010	03:08:07	0.9	31.744	-94.209	5100	W2016
02/08/2010	04:34:30	3.0	31.074	-91.1	37000	W2016
12/08/2010	19:00:21	1.2	31.884	-94.436	300	W2016
25/08/2010	19:35:01	2.7	30.86	-92.247	34400	W2016
29/08/2010	19:58:25	1.1	31.645	-93.745	500	W2016
29/08/2010	19:59:54	1.1	31.645	-93.745	500	W2016
30/08/2010	14:05:31	2.0	31.644	-93.744	500	W2016
30/08/2010	15:07:04	1.3	31.645	-93.745	500	W2016
30/08/2010	21:38:40	1.3	31.644	-93.745	500	W2016
30/08/2010	21:56:01	1.3	31.645	-93.744	500	W2016
10/10/2010	21:11:14	1.1	31.885	-94.436	300	W2016
01/12/2010	04:05:46	1.5	31.744	-94.21	5100	W2016
04/07/2011	02:36:35	2.0	31.901	-94.438	2000	W2016
03/08/2011	11:08:50	1.9	30.227	-92.023	62100	W2016
08/08/2011	09:18:11	0.5	32.361	-93.268	2600	W2016
13/08/2011	18:15:08	1.5	32.36	-93.268	2800	W2016

14/08/2011	21:53:37	0.8	32.36	-93.269	2700	W2016
15/08/2011	04:12:28	1.4	32.361	-93.268	2800	W2016
16/08/2011	00:56:31	0.7	32.361	-93.269	2700	W2016
16/08/2011	08:48:26	0.8	32.36	-93.268	2700	W2016
27/08/2011	03:53:54	0.7	32.361	-93.268	2700	W2016
05/09/2011	11:52:14	1.0	32.359	-93.227	5000	W2016
07/09/2011	03:24:45	0.9	32.36	-93.228	5000	W2016
07/09/2011	06:11:23	0.5	32.359	-93.227	5100	W2016
09/09/2011	04:48:34	1.3	32.359	-93.228	4900	W2016
09/09/2011	09:34:02	0.6	32.359	-93.228	5100	W2016
27/09/2011	06:39:43	1.4	32.361	-93.268	2800	W2016
27/09/2011	09:17:35	1.5	32.365	-93.273	7400	W2016
27/09/2011	09:25:17	0.7	32.364	-93.275	7400	W2016
14/10/2011	22:31:22	1.4	32.361	-93.268	2800	W2016
15/10/2011	10:55:41	1.9	32.365	-93.253	1300	W2016
29/10/2011	09:19:28	0.9	32.36	-93.268	2800	W2016
26/11/2011	21:22:13	2.1	31.887	-94.434	300	W2016
23/02/2012	01:21:03	1.6	31.886	-94.437	300	W2016
10/05/2012	15:15:40	3.9	31.92	-94.493	7600	W2016
11/05/2012	08:35:44	1.7	31.891	-94.573	35500	W2016
14/05/2012	07:37:07	1.5	31.886	-94.581	37600	W2016
17/05/2012	08:12:02	4.7	31.9	-94.476	3500	W2016
03/02/2013	06:40:56	2.1	31.545	-94.162	5000	ComCat
03/10/2014	04:52:32	3.1	31.676	-94.055	5000	ComCat
04/12/2018	07:14:13	2.4	32.351	-93.765	5000	ComCat
07/12/2021	03:20:00	2.1	32.535	-93.443	80	ComCat
04/09/2018	15:06:47	3.8	31.894	-94.481	8500	TexNet
12/09/2018	04:35:06	2.2	31.906	-94.457	6100	TexNet
09/11/2018	09:58:35	2.4	31.359	-94.275	6700	TexNet
10/11/2018	01:46:22	2.5	31.418	-94.284	4400	TexNet
22/11/2018	06:33:46	2.7	31.41	-94.3	6900	TexNet
26/11/2018	22:38:40	3.0	31.43	-94.315	5600	TexNet
26/11/2018	23:45:18	3.0	31.434	-94.298	6100	TexNet
27/11/2018	14:12:35	2.7	31.423	-94.307	6900	TexNet
28/11/2018	13:28:44	2.4	31.422	-94.209	10600	TexNet
02/12/2018	08:03:53	3.3	31.41	-94.3	6000	TexNet
14/12/2018	23:40:57	2.3	31.431	-94.293	5600	TexNet
15/12/2018	09:55:27	3.1	31.433	-94.306	9000	TexNet

16/12/2018	03:25:27	2.3	31.435	-94.286	9300	TexNet
17/12/2018	03:37:11	3.2	31.422	-94.306	7500	TexNet
17/12/2018	08:50:27	3.1	31.415	-94.308	6500	TexNet
30/12/2018	23:46:37	2.4	31.361	-94.288	4900	TexNet
01/01/2019	12:16:37	2.9	31.427	-94.282	7500	TexNet
20/01/2019	11:32:04	3.6	31.415	-94.297	5500	TexNet
28/01/2019	06:03:59	2.1	31.836	-94.445	9200	TexNet
02/02/2019	20:06:52	1.9	31.614	-93.999	9300	TexNet
16/03/2019	02:47:31	2.6	32.557	-94.949	12500	TexNet
20/04/2019	22:18:29	2.5	31.62	-94.05	3400	TexNet
27/07/2019	04:01:02	2.4	31.841	-94.434	6300	TexNet
12/10/2019	03:25:00	3.0	31.585	-94.811	6100	TexNet
23/03/2020	06:52:42	3.2	31.96	-94.432	6000	TexNet
15/04/2021	05:13:57	3.1	32.594	-93.999	16500	TexNet
16/04/2021	00:43:50	3.7	32.612	-94.04	11800	TexNet
29/12/2021	15:41:08	3.1	31.605	-94.792	7600	TexNet
30/12/2023	11:30:44	2.2	31.738	-93.967	9100	TexNet
30/12/2023	11:43:10	2.4	31.757	-93.984	4200	TexNet
31/12/2023	11:10:49	2.4	31.78	-93.97	4100	TexNet
02/04/2024	22:17:27	3.0	31.473	-94.404	7600	TexNet
04/05/2024	02:21:41	2.6	31.473	-94.398	6400	TexNet

Table S3: Template events and analysis periods for template matching for each event cluster. The template locations and magnitudes reported in this table correspond to the values reported in the regional catalogs.

Chireno 2018/2019

Analysis Period 2018/08/01 – 2019/06/01 **Station: TX.SNAG**

Templates:

Date	Time	Magnitude	Lat [°]	Lon [°]	Depth [m]
2019/01/20	11:32:04	3.6	31.414	-94.297	5500
2019/01/01	12:16:37	2.9	31.427	-94.282	7500
2018/12/30	23:46:37	2.4	31.361	-94.288	4900
2018/12/17	08:50:27	3.1	31.414	-94.308	6500
2018/12/17	03:37:11	3.2	31.422	-94.306	7500
2018/12/16	03:25:27	2.3	31.435	-94.286	9300
2018/12/15	09:55:27	3.1	31.433	-94.306	9000
2018/12/14	23:40:57	2.3	31.431	-94.293	5600
2018/12/02	08:03:53	3.3	31.410	-94.300	6000
2018/11/28	13:28:44	2.4	31.422	-94.209	10600
2018/11/27	14:12:35	2.7	31.423	-94.307	6900
2018/11/26	23:45:18	3.0	31.434	-94.298	6100
2018/11/26	22:38:40	3.0	31.430	-94.315	5600
2018/11/22	06:33:46	2.7	31.410	-94.300	6900
2018/11/10	01:46:22	2.5	31.418	-94.284	4400
2018/11/09	09:58:35	2.4	31.359	-94.274	6700

San Augustine 2019

Analysis Period: 2018/08/01 – 2019/06/01 **Station: TX.SNAG**

Templates:

Date	Time	Magnitude	Lat [°]	Lon [°]	Depth [m]
2019/04/20	22:18:29	2.5	31.617	-94.051	3400
2019/02/02	20:06:52	1.9	31.614	-94.000	9300

San Augustine 2023/24

Analysis Period: 2023/10/01 – 2024/03/01 **Station: TX.SNAG**

Analysis Period: 2023/12/01 – 2024/02/01 **Station: TX.ET01**

Templates:

Date	Time	Magnitude	Lat [°]	Lon [°]	Depth [m]
2019/04/20	22:18:29	2.5	31.617	-94.051	3400
2019/02/02	20:06:52	1.9	31.614	-94.000	9300
2023/12/30	11:30:44	2.2	31.738	-93.967	9100

2023/12/30	11:43:10	2.4	31.757	-93.984	4200
2023/12/31	11:10:49	2.4	31.780	-93.970	4100

Lake Nacogdoches

Analysis Period: 2019/08/01 – 2020/01/01 **Station: TX.ET01**

Analysis Period: 2021/10/01 – 2022/03/01 **Station: TX.ET01**

Templates:

Date	Time	Magnitude	Lat [°]	Lon [°]	Depth [m]
2021/12/29	15:41:08	3.1	31.605	-94.792	7600
2019/10/12	03:25:00	3.0	31.585	-94.812	6100

Table S4: Table of events detected and located in this study. The uncertainties in easting, northing and depth (ΔE, ΔN, ΔZ) give the half length of the uncertainty ellipsoid projected into each direction.

Date	Time	Mag	Lat [°]	Lon [°]	Depth [m]	ΔE [m]	ΔN [m]	ΔZ [m]
**Chireno Cluster**								
2018/11/09	09:57:55	2.1	31.431	-94.289	1233	116	131	174
2018/11/09	11:37:50	1.9	31.418	-94.279	6000	2848	1527	6768
2018/11/09	18:28:13	2.2	31.426	-94.285	2105	518	1168	1400
2018/11/10	01:45:41	2.3	31.441	-94.308	800	133	158	195
2018/11/16	14:25:49	2.4	31.478	-94.331	1600	877	1569	1160
2018/11/16	15:29:21	2.4	31.438	-94.292	399	698	933	800
2018/11/17	19:12:06	1.9	31.449	-94.298	1600	663	822	1521
2018/11/22	06:33:07	2.4	31.448	-94.289	3800	769	935	479
2018/11/26	22:38:01	2.7	31.459	-94.295	2872	411	806	1117
2018/11/26	23:44:38	2.8	31.455	-94.291	2915	745	949	1200
2018/11/27	14:11:55	2.6	31.456	-94.294	3801	381	781	544
2018/11/28	13:28:03	2.1	31.409	-94.280	4972	496	931	1171
2018/11/30	12:17:16	2.4	31.416	-94.277	950	134	142	185
2018/12/02	08:03:13	3.0	31.458	-94.297	1268	403	733	900
2018/12/14	23:40:17	2.0	31.457	-94.306	2206	516	798	1449
2018/12/15	09:54:47	2.8	31.465	-94.299	1600	423	766	1278
2018/12/16	03:24:47	2.1	31.460	-94.301	1440	406	681	1347
2018/12/17	03:36:30	3.0	31.446	-94.305	2283	502	773	1111
2018/12/17	08:49:47	2.7	31.462	-94.299	1402	430	594	899
2018/12/30	23:45:58	2.0	31.442	-94.295	3800	467	715	610
2019/01/01	12:15:57	2.7	31.462	-94.305	3800	432	795	370
2019/01/20	11:31:24	3.3	31.456	-94.298	2540	459	960	1172
2019/03/03	06:48:40	1.8	31.409	-94.319	3168	724	2443	2631
**San Augustine Clusters**								
2019/02/02	20:06:11	1.9	31.630	-94.028	1599	2196	1017	1664
2019/04/20	22:17:48	2.3	31.644	-94.010	925	305	247	306
2023/12/29	03:29:11	2.1	31.743	-94.062	1898	922	515	566
2023/12/30	05:54:43	1.6	31.740	-94.065	5201	2653	1522	3601
2023/12/30	11:30:05	2.0	31.741	-94.029	3800	1942	1403	3213
2023/12/30	11:42:31	2.2	31.737	-94.032	3800	1712	1261	3071
2023/12/31	11:10:10	2.2	31.736	-94.052	7000	1622	953	3246
2023/12/31	13:12:20	1.9	31.739	-94.031	3800	1973	1435	3100
**Lake Nacogdoches Cluster**								
2019/10/12	03:24:20	2.8	31.587	-94.767	3800	687	764	1085
2021/12/29	15:40:28	3.2	31.594	-94.763	4000	2005	1026	1553

Table S5: Table of events detected but with insufficient phases for a robust location. The positions reported here are based on waveform similarity with the template events, as per Equation 1.

Date	Time	Mag	Lat [°]	Lon [°]
**Chireno Cluster**				
2018/11/09	07:14:42	1.0	31.434	-94.291
2018/11/09	07:40:34	0.9	31.445	-94.292
2018/11/09	09:55:59	1.5	31.440	-94.291
2018/11/09	10:05:07	1.4	31.449	-94.296
2018/11/09	13:42:18	1.2	31.450	-94.299
2018/11/09	18:18:24	1.4	31.440	-94.291
2018/11/10	02:12:47	1.1	31.448	-94.295
2018/11/10	09:52:09	1.5	31.449	-94.296
2018/11/10	13:04:17	1.4	31.441	-94.308
2018/11/11	03:45:30	1.6	31.450	-94.296
2018/11/11	04:42:09	1.5	31.449	-94.297
2018/11/11	05:02:25	1.3	31.449	-94.296
2018/11/12	02:27:37	1.0	31.452	-94.299
2018/11/14	05:51:57	1.5	31.450	-94.297
2018/11/15	11:43:31	1.2	31.447	-94.295
2018/11/16	08:00:16	1.3	31.450	-94.299
2018/11/16	08:17:19	1.4	31.448	-94.296
2018/11/19	16:11:35	1.3	31.453	-94.297
2018/11/21	23:36:29	1.2	31.448	-94.296
2018/11/22	07:39:55	1.8	31.450	-94.297
2018/11/26	11:06:51	1.0	31.441	-94.308
2018/11/27	00:20:22	1.0	31.457	-94.299
2018/11/28	13:07:45	1.3	31.452	-94.297
2018/12/09	03:42:49	1.7	31.449	-94.296
2018/12/09	12:12:26	1.3	31.440	-94.291
2018/12/14	08:23:37	1.4	31.454	-94.299
2018/12/14	16:16:02	1.5	31.454	-94.303
2018/12/15	11:19:54	1.4	31.456	-94.302
2018/12/16	10:38:16	1.8	31.451	-94.298
2019/01/25	09:45:36	1.0	31.441	-94.291
2019/03/16	21:10:43	1.3	31.451	-94.297
**San Augustine Clusters**				
2019/01/15	10:12:20	1.9	31.633	-94.024
2019/04/23	23:27:56	1.1	31.644	-94.010
2023/12/24	00:05:56	1.4	31.738	-94.037

2023/12/28	13:36:38	1.7	31.738	-94.038
2023/12/29	04:10:47	1.6	31.738	-94.036
2023/12/29	04:17:45	1.2	31.741	-94.029
2023/12/30	11:31:02	1.3	31.738	-94.037
2024/01/01	04:39:03	1.2	31.738	-94.038
2024/01/04	12:18:06	1.8	31.738	-94.038
2024/01/09	07:35:26	1.4	31.738	-94.038

Table S6: Verdon et al. (2019) induced seismicity assessment framework applied to the 2018/2019 Chireno cluster. We consider two possible causes: hydraulic fracturing in wells 40530675, 40530671, and 40530672, and WWD in well 4234733181

**Question**	**Score**	**HF wells**<br>**EvW (%) | Answer**	**WWD well**<br>**EvW (%) | Answer**
**Q1. Has there been previous (either historical or instrumental) seismicity at the same site, or within the same regional setting?**		EW: 0 %	EW: 0 %
Earthquakes have previously occurred in vicinity to the site, with similar rates and magnitudes	-5	No data	No data
Earthquakes have previously occurred within the same regional setting, with similar rates and magnitudes	-2	No data	No data
Earthquakes have not occurred at similar rates or magnitudes within the regional setting	+5	No data	No data
Past earthquakes occurred at similar depths within the regional setting	-3	No data	No data
Earthquakes are significantly shallower than any past events that have been observed within the regional setting	+3	No data	No data
**Notes:** Eastern Texas has a relatively low background rate of seismicity, and historically, monitoring in the area has been of limited quality. The situation is further complicated by the fact that many previous earthquakes in Texas may be induced (Frohlich et al., 2016). The largest earthquake in the Chireno sequence is M_L 3.3. Natural earthquakes of this magnitude have previously occurred (Frohlich and Davis, 2003). What is notable about the Chireno sequence is that it consists of a burst of at least 54 events within the space of 3 months. The magnitudes of these events were mostly below M_L 2.5, and so therefore would likely have gone undetected prior to the installation of TexNet. We are therefore not able to assess whether this rate of activity, albeit at low magnitude, has previously occurred for natural events in Texas.			
**Q2. Is there temporal coincidence between the onset of events and the industrial activities?**		EW: 100 %	EW: 100 %
The earthquake sequence began prior to the commencement of industrial activity	-15	No	No
The earthquake sequence did not begin until a significant period of time after the cessation of industrial activity	-5	No	No
The earthquake sequence began while the industrial activity was ongoing	+5	**Yes**	**Yes**
**Notes:** The seismicity in this cluster began while both HF and WWD operations were ongoing.			
**Q3. Are the observed seismic events temporally correlated with the injection or extraction activities?**		EW: 100 %	EW: 100 %
The earthquakes are coincident with the industrial activity, but there is minimal correlation	-4	No	**Yes**

There is some temporal correlation between the seismicity and the industrial activity	+4	No	No
There is strong temporal correlation between the seismicity and the industrial activity (e.g., between rates of injection and rates of seismicity)	+15	**Yes**	No
**Notes:** There is strong temporal correlation between the HF operations and the seismicity, with the seismicity beginning shortly after HF operations began, and ceasing two weeks after operations stopped. The WWD has injected fluid fairly continuously since 2011 – there is coincidence, but no correlation between that injection and the seismicity.			
**Q4. Do the events occur at similar depths to the activities?**		EW: 80%	EW: 80 %
Earthquakes do not occur at the same depth, and there is no plausible mechanism by which stress or pressure changes could be transferred to these depths	-4	No	No
Earthquakes do not occur at the same depth, but plausible mechanisms exist by which stress or pressure changes could be transferred to these depths	+2	No	No
Earthquakes occur at similar depths to the industrial activity	+3	**Yes**	**Yes**
**Notes:** Depth uncertainties are generally between 2 – 3 km for these events. However, hypocentres do appear to be shallow (< 6 km). Hydraulic fracturing operations were at approximately 4,000 m depth, while WWD was at roughly 1,600 m depth.			
**Q5. Is there spatial collocation between events and the activities?**		EW: 100 %	EW: 50 %
Earthquakes are distant to the activities, given the putative causative mechanism	-10	No	No
Earthquakes are sufficiently close to the activities, given the putative causative mechanism	+5	**Yes**	**Yes**
If earthquake loci change with time, this change is consistent with the industrial activity, for example, growing radially from a well or shifting in response to the start of a new well	+10	**Yes**	No
**Notes:** Events are very close to the HF wells, clearly within sufficient proximity to be a cause. There is an apparent shift in the hypocentres to the NW during the sequence, which is consistent with a shift in HF operations from well 40530675 to wells 40530672 and 40530671. The events are approximately 7 – 8 km from the WWD well. High volume WWD has caused induced seismicity at these distances, but this would depend on the injected volumes and the size of the resulting pressure pulse, which has not been modelled in this study.			
**Q6. Is there a plausible mechanism to have caused the events?**		EW: 80 %	EW: 0 %
No significant pore-pressure increase or decrease occurred that can be linked in a plausible manner to the event hypocentral position	-5	No	No data
Some pore-pressure or poroelastic stress change occurred that can be linked in a plausible manner to the event hypocentral position	+2	No	No data

A large pore-pressure or poroelastic stress change occurred that can be linked in a plausible manner to the event hypocentral position	+5	**Yes**	No data
**Notes:** We do not have data as to the injection pressures at the HF wells. However, by definition, HF injection pressures must exceed the minimum stress. Given that the HF wells and the events overlap spatially, HF would therefore have generated high pressures at the positions of the events. The WWD is further away – without more detailed subsurface modelling it is not possible to establish whether the pressure pulse from WWD would have reached the position of the event hypocentres.			
**Q7. Do the source mechanisms indicate an induced event mechanism?**		EW: 0 %	EW: 0 %
The source mechanisms are consistent with the regional stress conditions	0	No data	No data
Source mechanisms are not consistent with the regional stress conditions, but are consistent with a putative causative mechanism (e.g., thrust faults above a subsiding reservoir)	+4	No data	No data
**Notes:** Given the relatively sparse recording array and low signal to noise ratios for these events, we have not computed focal mechanisms.			

Results

**Cause**	**Evidence Strength Ratio (ESR):**	**Induced Assessment Ratio (IAR):**
**HF Wells**	**76 %**	**+100 %**
**WWD Wells**	**57 %**	**+27 %**

Summary: The ESR values are moderate to high – the main aspect of the evidence that is lacking is information about historical rates of earthquakes with relatively low magnitudes ($M_L < 3.0$). For the WWD well, pressure simulations are needed to establish whether the perturbation could have extended 7 – 8 km to reach the position of the events. The IAR score for the HF wells is very high, indicating that these wells are extremely likely to be the cause of these events. The IAR score for the WWD is positive but low, indicating that this well could be a possible cause, but this finding is ambiguous. Given the strong positive IAR score for the HF wells, we conclude that this sequence of events has been induced by the identified HF wells.

Table S7: Verdon et al. (2019) induced seismicity assessment framework applied to the San Augustine 2019 cluster. We consider two possible causes: hydraulic fracturing in wells 41931792 and 41931794, and WWD in several nearby wells.

**Question**	**Score**	**HF wells**<br>**EvW (%) | Answer**	**WWD wells**<br>**EvW (%) | Answer**
**Q1. Has there been previous (either historical or instrumental) seismicity at the same site, or within the same regional setting?**		EW: 50 %	EW: 50 %
Earthquakes have previously occurred in vicinity to the site, with similar rates and magnitudes	-5	No	No
Earthquakes have previously occurred within the same regional setting, with similar rates and magnitudes	-2	**Yes**	**Yes**
Earthquakes have not occurred at similar rates or magnitudes within the regional setting	+5	No	No
Past earthquakes occurred at similar depths within the regional setting	-3	No data	No data
Earthquakes are significantly shallower than any past events that have been observed within the regional setting	+3	No data	No data
**Notes:** Eastern Texas has a relatively low background rate of seismicity, and historically, monitoring in the area has been of limited quality. The situation is further complicated by the fact that many previous earthquakes in Texas may be induced (Frohlich et al., 2016). The largest earthquake in the San Augustine sequence is M_L 2.3. Natural earthquakes larger than this magnitude have previously occurred (Frohlich and Davis, 2003). The sequence consists of 4 detected events over a period of 4 months, 3 of which had magnitudes less than M_L 2.0. It is likely that such rates have been exceeded during natural earthquake sequences in the region.			
**Q2. Is there temporal coincidence between the onset of events and the industrial activities?**		EW: 100 %	EW: 100 %
The earthquake sequence began prior to the commencement of industrial activity	-15	No	No
The earthquake sequence did not begin until a significant period of time after the cessation of industrial activity	-5	No	No
The earthquake sequence began while the industrial activity was ongoing	+5	**Yes**	**Yes**
**Notes:** Each event in this cluster occurred during HF operations in nearby wells, or shortly after. The WWD operations have been ongoing throughout the period of interest.			
**Q3. Are the observed seismic events temporally correlated with the injection or extraction activities?**		EW: 50 %	EW: 50 %
The earthquakes are coincident with the industrial activity, but there is minimal correlation	-4	No	**Yes**

There is some temporal correlation between the seismicity and the industrial activity	+4	**Yes**	No
There is strong temporal correlation between the seismicity and the industrial activity (e.g., between rates of injection and rates of seismicity)	+15	No	No
**Notes:** The low number of events in this sequence makes it challenging to robustly assess correlation. However, the events occurred during or immediately after hydraulic fracturing in the identified wells, and at no other times. WWD operations have injected fluid fairly continuously since 2011 – the absence of events at times other than when HF operations were occurring suggests that the seismicity is not temporally correlated with the WWD activities.			
**Q4. Do the events occur at similar depths to the activities?**		EW: 80%	EW: 80 %
Earthquakes do not occur at the same depth, and there is no plausible mechanism by which stress or pressure changes could be transferred to these depths	-4	No	No
Earthquakes do not occur at the same depth, but plausible mechanisms exist by which stress or pressure changes could be transferred to these depths	+2	No	No
Earthquakes occur at similar depths to the industrial activity	+3	**Yes**	**Yes**
**Notes:** Depth uncertainties are as much as 3 km for these events. However, hypocentres do appear to be shallow. Hydraulic fracturing operations were at approximately 4,000 m depth, while WWD took place at depths of between 800 – 2,100 m.			
**Q5. Is there spatial collocation between events and the activities?**		EW: 50 %	EW: 50 %
Earthquakes are distant to the activities, given the putative causative mechanism	-10	No	No
Earthquakes are sufficiently close to the activities, given the putative causative mechanism	+5	**Yes**	**Yes**
If earthquake loci change with time, this change is consistent with the industrial activity, for example, growing radially from a well or shifting in response to the start of a new well	+10	No	No
**Notes:** The location uncertainties for these events are relatively high. Given the uncertainties, the events could be placed in sufficient proximity to the identified HF wells for them to represent a plausible cause. The location uncertainties could also place the events within close proximity to the WWD wells. The size of the pressure pulse that may have been caused by the WWD wells has not been modelled in this study.			
**Q6. Is there a plausible mechanism to have caused the events?**		EW: 80 %	EW: 0 %
No significant pore-pressure increase or decrease occurred that can be linked in a plausible manner to the event hypocentral position	-5	No	No data
Some pore-pressure or poroelastic stress change occurred that can be linked in a plausible manner to the event hypocentral position	+2	**Yes**	No data

<table>
<tr><td>A large pore-pressure or poroelastic stress change occurred that can be linked in a plausible manner to the event hypocentral position</td><td>+5</td><td>No</td><td>No data</td></tr>
<tr><td colspan="4">Notes: We do not have data as to the injection pressures at the HF wells. However, by definition, HF injection pressures must exceed the minimum stress. HF could therefore have generated high pressures at the positions of the events. Without more detailed subsurface modelling it is not possible to establish the extent and magnitude of the pressure pulse from WWD.</td></tr>
<tr><td>Q7. Do the source mechanisms indicate an induced event mechanism?</td><td></td><td>EW: 0 %</td><td>EW: 0 %</td></tr>
<tr><td>The source mechanisms are consistent with the regional stress conditions</td><td>0</td><td>No data</td><td>No data</td></tr>
<tr><td>Source mechanisms are not consistent with the regional stress conditions, but are consistent with a putative causative mechanism (e.g., thrust faults above a subsiding reservoir)</td><td>+4</td><td>No data</td><td>No data</td></tr>
<tr><td colspan="4">Notes: We have not computed focal mechanisms for these events.</td></tr>
</table>

Results

Cause	Evidence Strength Ratio (ESR):	Induced Assessment Ratio (IAR):
**HF Wells**	**61 %**	**+47 %**
**WWD Wells**	**52 %**	**+31 %**

Summary: The ESR values are moderate – information about historical rates of earthquakes with relatively low magnitudes ($M_L < 3.0$) is lacking. The low number of events makes it difficult to establish whether correlation exists with the HF operations, and the location uncertainties make it difficult to establish whether they are in sufficient proximity to the active wells. For the WWD well, pressure simulations are needed to establish the magnitude and extent of the pressure perturbation. The IAR score for the HF wells is moderate and positive, indicating that these wells are likely to be the cause of these events, though combined with the moderate ESR score, this is not a foregone conclusion. The IAR score for the WWD wells is also positive but lower, indicating that this well could also be considered as a possible cause, but the HF wells are the more likely cause.

Table S8: Verdon et al. (2019) induced seismicity assessment framework applied to the San Augustine 2023/24 cluster. We consider two possible causes: hydraulic fracturing in wells 41931844, 41931845, 41931846, and 41931847, and WWD in well 41931048.

**Question**	**Score**	**HF wells**<br>**EvW (%) | Answer**	**WWD wells**<br>**EvW (%) | Answer**
**Q1. Has there been previous (either historical or instrumental) seismicity at the same site, or within the same regional setting?**		EW: 0 %	EW: 0 %
Earthquakes have previously occurred in vicinity to the site, with similar rates and magnitudes	-5	No data	No data
Earthquakes have previously occurred within the same regional setting, with similar rates and magnitudes	-2	No data	No data
Earthquakes have not occurred at similar rates or magnitudes within the regional setting	+5	No data	No data
Past earthquakes occurred at similar depths within the regional setting	-3	No data	No data
Earthquakes are significantly shallower than any past events that have been observed within the regional setting	+3	No data	No data
**Notes:** The largest earthquake in the San Augustine sequence is M_L 2.2. Natural earthquakes of this magnitude have previously occurred (Frohlich and Davis, 2003). The 2023/24 San Augustine sequence consists of a burst of at least 14 events within the space of a month. The magnitudes of these events were mostly below M_L 2.0, and so therefore would likely have gone undetected prior to the installation of TexNet. We are therefore not able to assess whether this rate of activity, albeit at low magnitude, has previously occurred for natural events in Texas.			
**Q2. Is there temporal coincidence between the onset of events and the industrial activities?**		EW: 100 %	EW: 100 %
The earthquake sequence began prior to the commencement of industrial activity	-15	No	No
The earthquake sequence did not begin until a significant period of time after the cessation of industrial activity	-5	No	No
The earthquake sequence began while the industrial activity was ongoing	+5	**Yes**	**Yes**
**Notes:** Each event in this cluster occurred during or HF operations in nearby wells. The WWD operations have been ongoing throughout the period of interest.			
**Q3. Are the observed seismic events temporally correlated with the injection or extraction activities?**		EW: 100 %	EW: 100 %
The earthquakes are coincident with the industrial activity, but there is minimal correlation	-4	No	**Yes**
There is some temporal correlation between the seismicity and the industrial activity	+4	No	No

There is strong temporal correlation between the seismicity and the industrial activity (e.g., between rates of injection and rates of seismicity)	+15	**Yes**	No
**Notes:** There is strong temporal correlation between the HF operations and the seismicity, with the seismicity beginning and ending during the period of HF operations. The WWD has injected fluid fairly continuously since 2011 – there is no correlation between that injection and the seismicity.			
**Q4. Do the events occur at similar depths to the activities?**		EW: 80%	EW: 80 %
Earthquakes do not occur at the same depth, and there is no plausible mechanism by which stress or pressure changes could be transferred to these depths	-4	No	No
Earthquakes do not occur at the same depth, but plausible mechanisms exist by which stress or pressure changes could be transferred to these depths	+2	No	No
Earthquakes occur at similar depths to the industrial activity	+3	**Yes**	**Yes**
**Notes:** Depth uncertainties are relatively high for these events. However, hypocentres do appear to be shallow (< 7 km). Hydraulic fracturing operations were at approximately 4,000 m depth, while WWD was at depths of between 1,000 – 2,100 m.			
**Q5. Is there spatial collocation between events and the activities?**		EW: 100 %	EW: 0 %
Earthquakes are distant to the activities, given the putative causative mechanism	-10	No	No data
Earthquakes are sufficiently close to the activities, given the putative causative mechanism	+5	**Yes**	No data
If earthquake loci change with time, this change is consistent with the industrial activity, for example, growing radially from a well or shifting in response to the start of a new well	+10	No	No data
**Notes:** The event locations directly overlap with the HF wells. However, the HF wells are closely spaced, such that there is no evidence within the location uncertainties of lateral migration with the locus of HF operations. There is a significant distance to the WWD (> 10 km). WWD-induced seismicity has occurred over such distances but would require transfer of pore pressure or stress perturbations over considerable distances. Detailed reservoir modelling would be required to demonstrate that this would be feasible.			
**Q6. Is there a plausible mechanism to have caused the events?**		EW: 100 %	EW: 0 %
No significant pore-pressure increase or decrease occurred that can be linked in a plausible manner to the event hypocentral position	-5	No	No data
Some pore-pressure or poroelastic stress change occurred that can be linked in a plausible manner to the event hypocentral position	+2	No	No data
A large pore-pressure or poroelastic stress change occurred that can be	+5	**Yes**	No data

linked in a plausible manner to the event hypocentral position			
**Notes:** We do not have data as to the injection pressures at the HF wells. However, by definition, HF injection pressures must exceed the minimum stress. Given that the HF wells and the events overlap spatially, HF would therefore have generated high pressures at the positions of the events. The WWD is further away – without more detailed subsurface modelling it is not possible to establish whether the pressure pulse from WWD would have reached the position of the event hypocentres.			
**Q7. Do the source mechanisms indicate an induced event mechanism?**		EW: 0 %	EW: 0 %
The source mechanisms are consistent with the regional stress conditions	0	No data	No data
Source mechanisms are not consistent with the regional stress conditions, but are consistent with a putative causative mechanism (e.g., thrust faults above a subsiding reservoir)	+4	No data	No data
**Notes:** We have not computed focal mechanisms for these events.			

Results

Cause	Evidence Strength Ratio (ESR):	Induced Assessment Ratio (IAR):
**HF Wells**	**78 %**	**+87 %**
**WWD Wells**	**46 %**	**+15 %**

Summary: The ESR values are moderate to high – the main aspect of the evidence that is lacking is information about historical rates of earthquakes with relatively low magnitudes ($M_L < 3.0$). For the WWD well, pressure simulations are needed to establish whether the perturbation could have extended to reach the position of the events, especially given the large distance between the events and the only active WWD well in the area. The IAR score for the HF wells is very high, indicating that these wells are extremely likely to be the cause of these events. The IAR score for the WWD is positive but low, indicating that the findings with respect to the WWD are ambiguous. Given the strong positive IAR score for the HF wells, we conclude that this sequence of events has been induced by the identified HF wells.

Table S9: Verdon et al. (2019) induced seismicity assessment framework applied to the Lake Nacogdoches cluster

**Question**	**Score**	**WWD wells**<br>**EvW (%) | Answer**
**Q1. Has there been previous (either historical or instrumental) seismicity at the same site, or within the same regional setting?**		EW: 50 %
Earthquakes have previously occurred in vicinity to the site, with similar rates and magnitudes	-5	No
Earthquakes have previously occurred within the same regional setting, with similar rates and magnitudes	-2	**Yes**
Earthquakes have not occurred at similar rates or magnitudes within the regional setting	+5	No
Past earthquakes occurred at similar depths within the regional setting	-3	No data
Earthquakes are significantly shallower than any past events that have been observed within the regional setting	+3	No data
**Notes:** Eastern Texas has a relatively low background rate of seismicity, and historically, monitoring in the area has been of limited quality. The situation is further complicated by the fact that many previous earthquakes in Texas may be induced (Frohlich et al., 2016). The largest earthquake in the Lake Nacogdoches sequence was M_L 3.2. Natural earthquakes of this magnitude have previously occurred (Frohlich and Davis, 2003). The Lake Nacogdoches sequences consists of only 2 detected events over a period of more than 2 years. It is likely that such rates have been exceeded during natural earthquake sequences in the region.		
**Q2. Is there temporal coincidence between the onset of events and the industrial activities?**		EW: 100 %
The earthquake sequence began prior to the commencement of industrial activity	-15	No
The earthquake sequence did not begin until a significant period of time after the cessation of industrial activity	-5	No
The earthquake sequence began while the industrial activity was ongoing	+5	**Yes**
**Notes:** The seismicity in this cluster began while WWD operations were ongoing.		
**Q3. Are the observed seismic events temporally correlated with the injection or extraction activities?**		EW: 100 %
The earthquakes are coincident with the industrial activity, but there is minimal correlation	-4	**No**
There is some temporal correlation between the seismicity and the industrial activity	+4	No

There is strong temporal correlation between the seismicity and the industrial activity (e.g., between rates of injection and rates of seismicity)	+15	Yes
**Notes:** The WWD has injected fluid fairly continuously since the mid 2000s, whereas only two events have been recorded, in 2019 and 2022. There is no correlation between that injection and the WWD rates.		
**Q4. Do the events occur at similar depths to the activities?**		EW: 80%
Earthquakes do not occur at the same depth, and there is no plausible mechanism by which stress or pressure changes could be transferred to these depths	-4	No
Earthquakes do not occur at the same depth, but plausible mechanisms exist by which stress or pressure changes could be transferred to these depths	+2	No
Earthquakes occur at similar depths to the industrial activity	+3	**Yes**
**Notes:** Depth uncertainties are relatively high for these events. However, hypocentres do appear to be shallow (< 5 km). Hydraulic fracturing operations were at approximately 4,000 m depth, while WWD was at depths between 1,600 – 2,800 m.		
**Q5. Is there spatial collocation between events and the activities?**		EW: 50 %
Earthquakes are distant to the activities, given the putative causative mechanism	-10	No
Earthquakes are sufficiently close to the activities, given the putative causative mechanism	+5	**Yes**
If earthquake loci change with time, this change is consistent with the industrial activity, for example, growing radially from a well or shifting in response to the start of a new well	+10	No
**Notes:** The events are approximately 8 km from the nearest WWD well. High volume WWD has caused induced seismicity at these distances, but this would depend on the injected volumes and the size of the resulting pressure pulse, which has not been modelled in this study.		
**Q6. Is there a plausible mechanism to have caused the events?**		EW: 0 %
No significant pore-pressure increase or decrease occurred that can be linked in a plausible manner to the event hypocentral position	-5	No data
Some pore-pressure or poroelastic stress change occurred that can be linked in a plausible manner to the event hypocentral position	+2	No data
A large pore-pressure or poroelastic stress change occurred that can be linked in a plausible manner to the event hypocentral position	+5	Yes data

**Notes:** Without more detailed subsurface modelling it is not possible to establish the extent and magnitude of the pressure pulse from WWD.		
**Q7. Do the source mechanisms indicate an induced event mechanism?**		EW: 0 %
The source mechanisms are consistent with the regional stress conditions	0	No data
Source mechanisms are not consistent with the regional stress conditions, but are consistent with a putative causative mechanism (e.g., thrust faults above a subsiding reservoir)	+4	No data
**Notes:** We have not computed focal mechanisms for these events.		

Results

Cause	Evidence Strength Ratio (ESR):	Induced Assessment Ratio (IAR):
**WWD Wells**	**62 %**	**+16 %**
**Summary:** The ESR values are moderate – the main aspect of the evidence that are lacking is information about historical rates of earthquakes with relatively low magnitudes ($M_L < 3.0$). Pressure simulations are needed to establish whether the perturbations from the WWD wells could have extended to reach the position of the events. The IAR score for WWD is positive but low, indicating that this activity could be a possible cause, but this finding is ambiguous.		

S10: Investigation of the Caddo Lake cluster

The Caddo Lake cluster is located at the northern edge of the Haynesville play area, on the border between Texas and Louisiana. It consists of two events reported in the TexNet catalog in April 2021. The larger event has a magnitude of M_L 3.7, making it the largest event of those investigated in our study.

We have not investigated this sequence in detail because it falls within the area covered by the Induced Seismicity in Louisiana (ISLA) temporary seismic monitoring array (Kraus et al., 2021), data from which is not currently available publicly. In contrast, this cluster falls outside the array of stations used in this study (see Figure 1). Robust detection of additional events and accurate relocations would require access to the ISLA dataset. We note that Kraus et al. (2021) reports additional events to those identified in the TexNet catalog for this cluster. Figure S11 shows a map and timeline of the events, and in Table S12 we use the Verdon et al. (2019) framework to evaluate whether the events were induced by nearby HF and WWD activities. HF activities in the Haynesville take place at roughly 3,400 m depths in this area, while the WWD wells are at depths of less than 1,000 m.

Given the spatial and temporal overlap between these events and the nearby HF operations, we find it likely that these events were caused by HF. However, further analysis using data from the Louisiana induced seismicity monitoring array would be useful in identifying more events within this sequence (and thereby further testing the temporal correlation between the events and the HF operations) and providing more accurate locations (thereby further investigating the spatial overlap between HF operations and the wells).

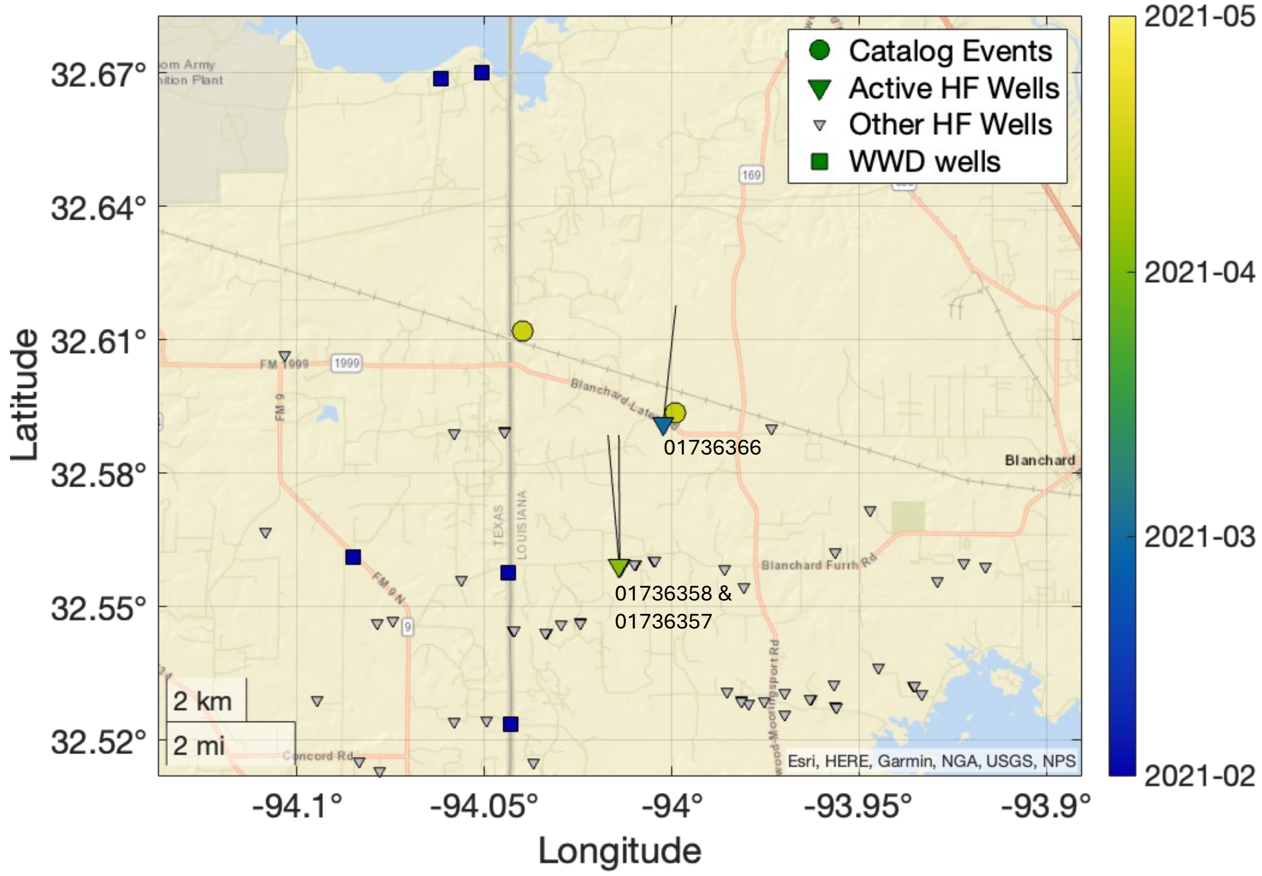

(a)

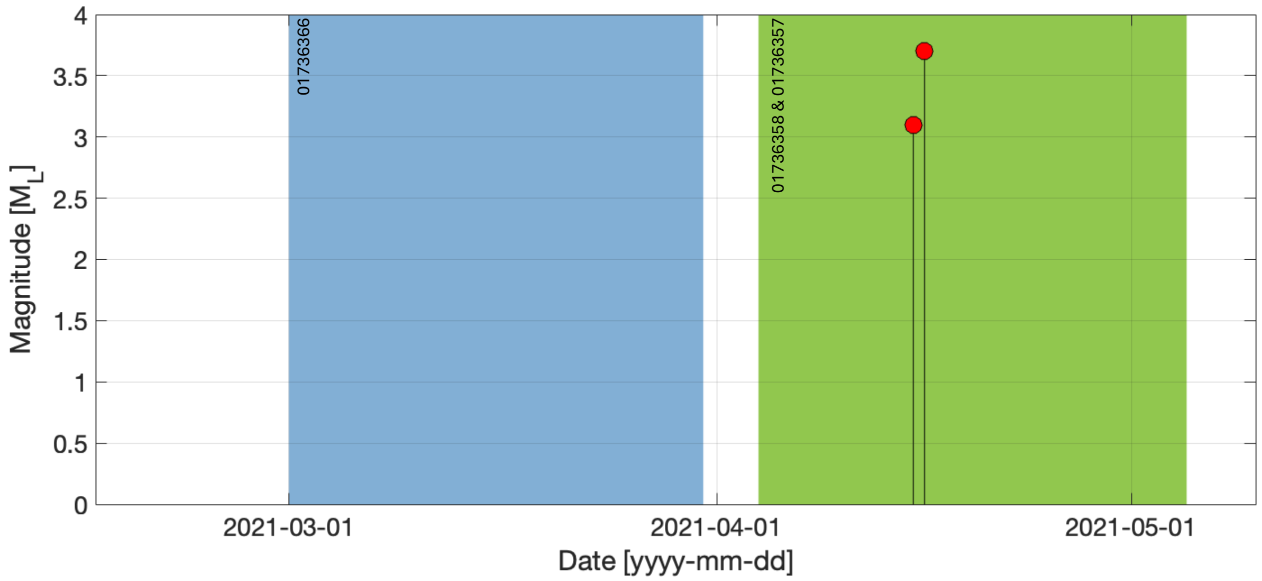

(b)

Figure S11: Map (a) and timeline (b) of earthquakes and hydraulic fracturing operations for the 2021 Caddo Lake cluster. Figure formats are as per Figure 3. Note that WWD wells are only shown for Texas, but similar numbers of WWD wells are found on the Louisiana side of the border.

Table S12: Verdon et al. (2019) induced seismicity assessment framework applied to the Caddo Lake cluster. We consider two possible causes: hydraulic fracturing in wells 01736366, 01736358 and 01736357, and WWD in several nearby wells.

Question	Score	HF wells<br>EvW (%) | Answer	WWD wells<br>EvW (%) | Answer
**Q1. Has there been previous (either historical or instrumental) seismicity at the same site, or within the same regional setting?**		EW: 0 %	EW: 0 %
Earthquakes have previously occurred in vicinity to the site, with similar rates and magnitudes	-5	No data	No data
Earthquakes have previously occurred within the same regional setting, with similar rates and magnitudes	-2	No data	No data
Earthquakes have not occurred at similar rates or magnitudes within the regional setting	+5	No data	No data
Past earthquakes occurred at similar depths within the regional setting	-3	No data	No data
Earthquakes are significantly shallower than any past events that have been observed within the regional setting	+3	No data	No data
**Notes:** The larger earthquake in the Caddo Lake sequence is M_L 3.7, and the sequence consists of two $M_L > 3.0$ events within two days. Natural earthquakes of this magnitude have previously occurred (Frohlich and Davis, 2003). With only two events, it is not possible to judge whether this sequence represents an elevation in earthquake rates above natural conditions.			
**Q2. Is there temporal coincidence between the onset of events and the industrial activities?**		EW: 100 %	EW: 100 %
The earthquake sequence began prior to the commencement of industrial activity	-15	No	No
The earthquake sequence did not begin until a significant period of time after the cessation of industrial activity	-5	No	No
The earthquake sequence began while the industrial activity was ongoing	+5	**Yes**	**Yes**
**Notes:** Each event in this cluster occurred during or HF operations in nearby wells. The WWD operations have been ongoing throughout the period of interest.			
**Q3. Are the observed seismic events temporally correlated with the injection or extraction activities?**		EW: 100 %	EW: 100 %
The earthquakes are coincident with the industrial activity, but there is minimal correlation	-4	No	**Yes**
There is some temporal correlation between the seismicity and the industrial activity	+4	No	No

There is strong temporal correlation between the seismicity and the industrial activity (e.g., between rates of injection and rates of seismicity)	+15	**Yes**	No
**Notes:** There is strong temporal correlation between the HF operations and the seismicity, with the seismicity beginning and ending during the period of HF operations. The WWD well has injected fluid fairly continuously for decades – there is no correlation between that injection and the seismicity.			
**Q4. Do the events occur at similar depths to the activities?**		EW: 50%	EW: 50 %
Earthquakes do not occur at the same depth, and there is no plausible mechanism by which stress or pressure changes could be transferred to these depths	-4	No data	No data
Earthquakes do not occur at the same depth, but plausible mechanisms exist by which stress or pressure changes could be transferred to these depths	+2	No data	No data
Earthquakes occur at similar depths to the industrial activity	+3	No	No
**Notes:** The catalog depths for these events are > 10 km. Depth uncertainties are reported as being less than 3 km, though our view is that this likely represents an underestimate of the true uncertainty. We do not have any data regarding faults or other potential conduits that could transfer pressures to greater depths. The HF in this area is at depths of roughly 3,400 m, while WWD is at depths of less than 1,000 m.			
**Q5. Is there spatial collocation between events and the activities?**		EW: 50 %	EW: 100 %
Earthquakes are distant to the activities, given the putative causative mechanism	-10	No	No
Earthquakes are sufficiently close to the activities, given the putative causative mechanism	+5	**Yes**	**Yes**
If earthquake loci change with time, this change is consistent with the industrial activity, for example, growing radially from a well or shifting in response to the start of a new well	+10	No	No
**Notes:** The location uncertainties for these events are relatively high. Given the uncertainties, the events could be placed in close proximity to the identified HF wells. The size of the pressure pulse that may have been caused by the WWD wells has not been modelled in this study, but given the number of WWD wells surrounding the events, some wells are likely to be in sufficient proximity to be plausible as a cause.			
**Q6. Is there a plausible mechanism to have caused the events?**		EW: 80 %	EW: 80 %
No significant pore-pressure increase or decrease occurred that can be linked in a plausible manner to the event hypocentral position	-5	No	No
Some pore-pressure or poroelastic stress change occurred that can be linked in a plausible manner to the event hypocentral position	+2	No	**Yes**
A large pore-pressure or poroelastic stress change occurred that can be	+5	**Yes**	No

linked in a plausible manner to the event hypocentral position			
**Notes:** We do not have data as to the injection pressures at the HF wells. However, by definition, HF injection pressures must exceed the minimum stress. The HF wells and the events could overlap spatially (given the location uncertainties), and HF could therefore have generated high pressures at the positions of the events. We do not have detailed subsurface modelling of the pressure perturbations caused by the WWD, but it is reasonable to surmise that some pressure perturbation from these wells could reach the positions of the events.			
**Q7. Do the source mechanisms indicate an induced event mechanism?**		EW: 0 %	EW: 0 %
The source mechanisms are consistent with the regional stress conditions	0	No data	No data
Source mechanisms are not consistent with the regional stress conditions, but are consistent with a putative causative mechanism (e.g., thrust faults above a subsiding reservoir)	+4	No data	No data
**Notes:** We have not computed focal mechanisms for these events.			

Results

Cause	Evidence Strength Ratio (ESR):	Induced Assessment Ratio (IAR):
**HF Wells**	**61 %**	**+87 %**
**WWD Wells**	**71 %**	**+21 %**

Summary: The ESR values are moderate to high – the main aspect of the evidence that is lacking is information about historical rates of earthquakes with relatively low magnitudes. For the WWD well, pressure simulations are needed to establish whether the perturbation could have extended to reach the position of the events, especially given the large distance between the events and the only active WWD well in the area. The IAR score for the HF wells is very high, indicating that these wells are likely to be the cause of these events. The IAR score for the WWD is positive but low, indicating that the findings with respect to the WWD are ambiguous. Given the strong positive IAR score for the HF wells, we conclude that this sequence of events has been induced by the identified HF wells.
